# Glycomic Signatures of Plasma IgG Improve Preoperative Prediction of the Invasiveness of Small Lung Nodules

**DOI:** 10.3390/molecules25010028

**Published:** 2019-12-20

**Authors:** Xia Zou, Feng Yao, Fang Yang, Fang Zhang, Zhijue Xu, Jingjing Shi, Atsushi Kuno, Heng Zhao, Yan Zhang

**Affiliations:** 1 Key Laboratory of Systems Biomedicine (Ministry of Education), Shanghai Center for Systems Biomedicine, Shanghai Jiao Tong University, Shanghai 200240, China; zouxia0206@sjtu.edu.cn (X.Z.); fangyang2011@sjtu.edu.cn (F.Y.); m13661409549@163.com (F.Z.); xuzhijue@gmail.com (Z.X.); shijingjing0813@163.com (J.S.); 2 Department of Thoracic Surgery, Shanghai Chest Hospital, Shanghai Jiao Tong University, Shanghai 200030, China; feng.yao@shchest.org (F.Y.); zh148@shchest.org (H.Z.); 3 Biotechnology Research Institute for Drug Discovery, National Institute of Advanced Industrial Science and Technology (AIST), Tsukuba 305-8568, Japan; atsu-kuno@aist.go.jp

**Keywords:** lectin microarray, multilectin assay, glycobiomarker, immunoglobulin G (IgG), ground glass nodule (GGN)

## Abstract

Preoperative assessment of tumor invasiveness is essential to avoid overtreatment for patients with small-sized ground-glass nodules (GGNs) of 10 mm or less in diameter. However, it is difficult to determine the pathological state by computed tomography (CT) examination alone. Aberrant glycans has emerged as a tool to identify novel potential disease biomarkers. In this study, we used a lectin microarray-based strategy to investigate whether glycosylation changes in plasma immunoglobulin G (IgG) provide additional information about the invasiveness of small GGNs before surgery. Two independent cohorts (discovery set, *n* = 92; test set, *n* = 210) of GGN patients were used. Five of 45 lectins (*Sambucus nigra* agglutinin, SNA; *Datura stramonium* agglutinin, DSA; *Galanthus nivalis* agglutinin, GNA; *Euonymus europaeus* lectin, EEL; and *Vicia villosa* agglutinin, VVA) were identified as independent factors associated with pathological invasiveness of small GGNs (*p* < 0.01). Receiver-operating characteristic (ROC) curve analysis indicated the combination of these five lectins could significantly improve the accuracy of CT in diagnosing invasive GGNs, with an area under the curve (AUC) of 0.792 (*p* < 0.001), a sensitivity of 74.6%, and specificity of 74.4%, which was superior to current clinical biomarkers. These results suggest that the multilectin assay based on plasma IgG glycosylation may be a useful in vitro complementary test to enhance preoperative determination of the invasiveness of GGNs and guide surgeons to select proper clinical management to avoid overtreatment.

## 1. Introduction

Lung cancer has the highest cancer incidence and is the highest cause of mortality worldwide, with an estimated 1.8 million deaths in 2018 [1]. In recent years, with the widespread acceptance of low-dose spiral computed tomography (LDCT) in clinical practice, the detection of solitary pulmonary nodules has increased markedly in China [2], of which over 54% are ground glass nodules (GGNs) (undisclosed data from Shanghai Chest Hospital). GGNs are defined as focal areas of increased CT attenuation within which the normal lung parenchyma structures, airways, and vessels can be observed [3]. These nodules can be diagnosed as focal fibrosis, noninvasive lesion including atypical adenomatous hyperplasia (AAH) and adenocarcinoma *in situ* (AIS), minimally invasive adenocarcinoma (MIA), or invasive adenocarcinoma (IA) according to their pathologic features [4]. As noninvasive lesions may remain unchanged and can be managed with close follow-up alone or safely treated with limited resection [5,6], extensive surgical resection of noninvasive lesions can cause unnecessary injuries to patients. Therefore, it is important to distinguish noninvasive lesions from invasive pulmonary adenocarcinomas before surgery so that the surgeon can select eligible patients for resection to avoid overtreatment.

Although increasing numbers of recent studies have reported distinguishing invasive GGNs by the visual assessment of CT imaging [7,8,9], there is still no unified consensus on the relationship between CT features and pathologic types of GGNs. In addition, some studies have also shown that pathologic characteristics in tumor tissues, such as the epidermal growth factor receptor (EGFR) mutation, human epidermal growth factor receptor type 3 (HER3), were differentially expressed during the progression of GGN from carcinoma *in situ* to invasive carcinoma [10,11,12]. However, these indications are limited by the nature of invasive detection. Thus, the indications for surgical resection of GGNs, especially small-sized GGNs ≤ 10 mm in diameter, remain controversial and complex. There is a critical need for the discovery of reliable blood-based indicators that can assist current CT examination to accurately predict the invasiveness of GGNs before surgery, which will significantly contribute to the reduction of overtreatment and benefit GGN patients with noninvasive lesions. 

Glycosylation is among the most common and fundamental post-translational protein modifications. Changes in glycosylation can significantly modulate the structure, stability, and function of glycoproteins, and these are closely associated with the pathological states of cells [13]. Therefore, aberrant glycosylation is widely observed in numerous human diseases, including cancer [14]. Currently, glycosylation-based biomarkers have emerged as promising candidates for the early detection, staging, and prognosis of cancer [15]. In particular, core fucosylation of α-fetoprotein (AFP-L3) greatly improved the diagnostic specificity of AFP in liver cancer [16]. However, evading immune destruction is considered an emerging hallmark of cancer [17]. Immunoglobin G (IgG), the most abundant glycoprotein in blood, is closely correlated with immune status. Recent studies have indicated the importance of altered glycosylation patterns of IgG in autoimmune diseases, infectious diseases, and different types of cancer [18,19,20]. Although several studies have reported declining levels of galactosylated N-glycans and bisecting GlcNAc structures on IgG in lung cancer [21,22,23], no studies have investigated the relationship between IgG glycosylation and pathological staging of small-sized pulmonary nodules.

In this study, we used a lectin microarray strategy to generate glycan signatures of IgG for GGNs at different pathological stages. We investigated whether the glycosylation profiles of plasma IgG were altered during the invasion process of GGNs and identified potential indicators that might assist CT imaging to accurately differentiate invasive GGNs before surgery.

## 2. Results

### 2.1. Patient Characteristics

**Figure 1:**
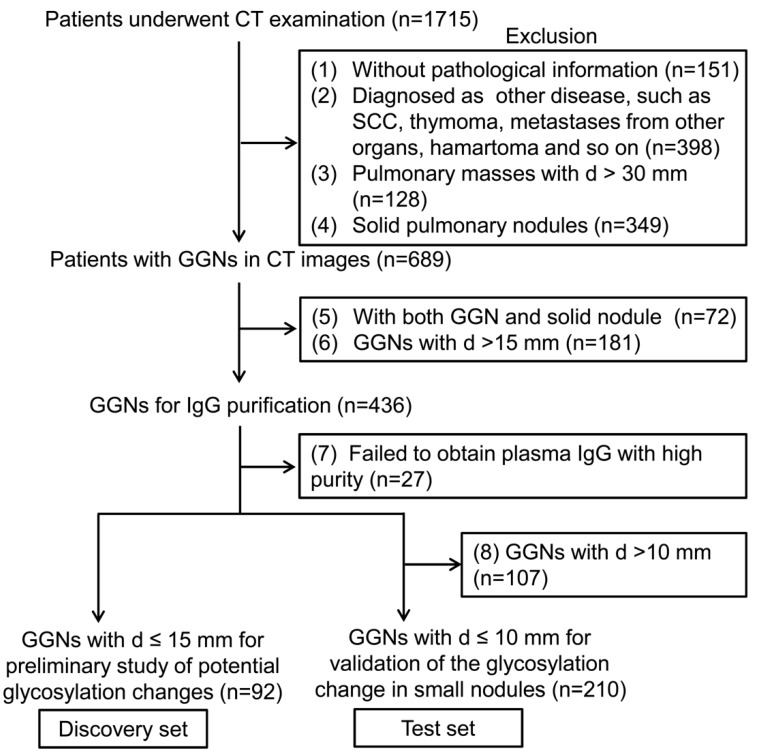
The flow diagram of the study population in this study. GGN, ground glass nodule; SCC, squamous cell carcinoma; d, diameter.

In this study, a total of 302 patients were used for lectin microarray analysis. Ninety-two specimens collected between January 2015 and September 2015 were considered as the discovery set for the preliminary search of potential glycosylation changes related to GGN invasiveness. Moreover, to validate specific glycosylation changes in small pulmonary nodules, 210 specimens ≤ 10 mm in diameter were used as the test set. Of note, sample preparation and analysis of the two sample sets were performed independently with a 1-year interval. A detailed patient inclusion flowchart is shown in Figure 1. 

The general characteristics of these 302 patients are summarized in Table 1. The study population consisted of 68 males (22.5%) and 234 females (77.5%), with a mean age of 52 years and a mean nodule size of 8.3 mm. Age and sex were matched between the noninvasive and invasive groups in both sets. In addition, CT values showed the most significant differences between the noninvasive and invasive groups in both the discovery set (*p* = 0.007) and test set (*p* = 0.001) while all other routine serological indicators, including CEA, Cyfra21-1, SCC-Ag, SCE, and CA125, did not differ significantly between these two groups. This suggests that the development of new serological indicators assisting CT examination is needed.

### 2.2. Purification of IgG from Plasma

In this study, we adopted a lectin microarray-based strategy to discover potential glycobiomarkers for invasive GGNs (Supplementary Figure S1). First, IgG was isolated from a small amount of plasma (0.5 μL) using Protein G magnetic beads. The procedures, including the incubation time, were optimized, and the efficiency and purity of obtained IgG fractions were analyzed by silver staining. As shown in Supplementary Figure S2A, the IgG elution presented as two bands between 55 and 25 kDa, which corresponded to the heavy and light chains of IgG. Moreover, because no significant difference in the amount of IgG elution was observed between different incubation times (5 min, 30 min, or 1 h), the incubation time used was 5 min to improve the throughput. Additionally, there was no significant difference in IgG bands between the duplicates of one sample, (① and ②), which indicates the purification process had good reproducibility. Of note, there was still a large amount of IgG in the flow through (FT) fractions, which indicates that the purification of IgG from 0.5 μL plasma using 10 μL magnetic beads was saturated. Thus, the amount of IgG enriched from each sample from patients with GGN is theoretically equal, which ensured the accuracy of the subsequent analysis by lectin microarray.

Based on the optimized procedures, plasma IgG was purified from 436 GGNs with diameter ≤ 15 mm. The representative silver staining of the IgG elution fraction is shown in Supplementary Figure S2B. However, we failed to obtain high IgG purity in 27 samples, which were excluded from further analysis.

### 2.3. IgG Glycosylation Changes Detected by Lectin Microarray

To detect the glycopattern of plasma IgG, we used a commercial lectin microarray (LecChip) which enables the ultrasensitive detection of most common glycan variants in mammals, including fucose, sialic acid, galactose, mannose, and *O*-glycan, by 45 lectins (Supplementary Table S1). First, we analyzed the linear response ranges of positive lectin spots (signal intensity > 1000) in a serial dilution of IgG elution fractions (10, 5, 2.5, 1.25, 0.625, and 0.313 μL) to determine the optimized concentration of IgG for lectin microarray (Supplementary Figure S3A). As shown in Supplementary Figure S3B, although most positive lectins showed dose-dependent increments of signal intensity from 0.313 to 10 μL, the positive rates of lectins were only 58% (26/45) under lower gains (Gain 75, 85, and 95). By contrast, the ratios of positive lectin spots increased under higher gains, such as 67% (30/45) at Gain105, 80% (36/45) at Gain115, and 87% (39/45) at Gain125. However, the linear response ranges of more positive spots became narrower. For example, the signals of nine positive lectins reached saturation at 5 μL or lower under Gain 105 while over 50% of positive lectins (19/39) did not show satisfactory linearity above 1.25 μL under Gain 125. Taken together, the amount of IgG elution fraction for lectin microarray was determined to be 2.5 μL, which ensured more lectins were positive and that the intensities of most positive spots were within an acceptable dynamic range (from 1000 to 40,000) [24]. Moreover, we evaluated the reproducibility of lectin microarray analysis. Six aliquots of IgG elution from one patient (#393) were subjected to six LecChips from different array lots. The CV values of the positive lectins were less than 20% (the manufacturer’s QC criteria) (Supplementary Figure S4). The correlation analysis showed that Pearson correlation coefficients of the 45 lectin signals between different chips were all larger than 0.985 (Supplementary Table S2), which also ensured a good reproducibility of the lectin microarray analysis.

**Figure 2:**
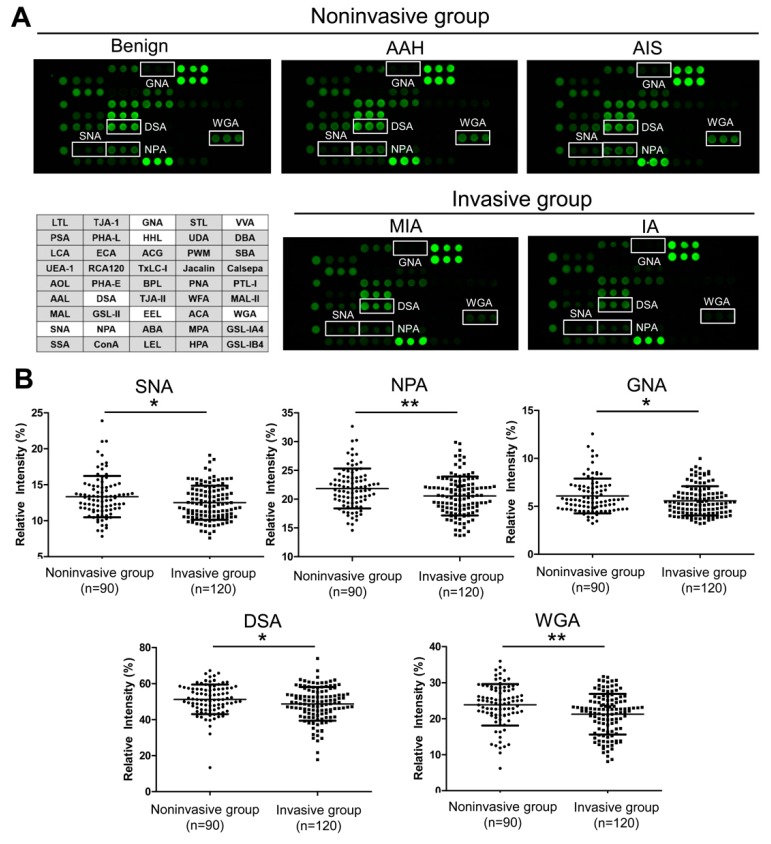
Differentially expressed IgG glycopatterns between noninvasive and invasive GGNs detected by lectin microarray. (**A**) The layout of the lectin microarray and representative array profiling of plasma IgG from noninvasive (benign, AAH, and AIS) and invasive (MIA and IA) GGN patients are shown. Differential signal patterns of eight lectins between the two groups are indicated in the open-box illustration in the array layout. Five lectins with signal > 1000 are marked with white frames in the representative profiling images. (**B**) The comparison of intensities of the five lectin signals (SNA, DSA, NPA, GNA, and WGA) between noninvasive (*n* = 90) and invasive (*n* = 120) GGNs less than 10 mm. * *p* < 0.05 and ** *p* < 0.01 from the Mann–Whitney U test. IgG, immunoglobin G; GGN, ground glass nodule; AAH, atypical adenomatous hyperplasia; AIS, adenocarcinoma *in situ*; MIA, minimally invasive adenocarcinoma; IA, invasive adenocarcinoma; SNA, *Sambucus nigra* agglutinin; DSA, *Datura stramonium* agglutinin; NPA, *Narcissus pseudonarcissus* agglutinin; GNA, *Galanthus nivalis* agglutinin; WGA, *Weat germ* agglutinin.

Based on these optimized conditions, we detected the glycan profiles of plasma IgG from 302 GGN patients, including the relatively independent discovery set (noninvasive group, *n* = 37; invasive group, *n* = 55) and test set (noninvasive group, *n* = 90; invasive group, *n* = 120). The discovery set was used for a preliminary screen of potential glycosylation features, and the test set was used for the further validation and discovery of the specific differential glycosylation features in small GGNs less than 10 mm in diameter. The representative glycosylation profiling images of GGNs at different pathological stages are shown in Figure 2A. After normalizing the lectin signals, 17 lectins of the 45-lectin array showed differential intensities between the noninvasive and invasive groups in the discovery set (*p* < 0.05) (Supplementary Table S3). Of these, eight lectins (*Sambucus nigra* agglutinin, SNA; *Datura stramonium* agglutinin, DSA; *Narcissus pseudonarcissus* agglutinin, NPA; *Galanthus nivalis* agglutinin, GNA; *Hippeastrum hybrid* lectin, HHL; *Euonymus europaeus* lectin, EEL; *Vicia villosa* agglutinin, VVA; and *Weat germ* agglutinin, WGA) were also verified as significant in the test set (Supplementary Table S3 and Table 2), of which five lectins (i.e., SNA, DSA, NPA, GNA, and WGA) were within the dynamic range (from 1000 to 40,000). Figure 2B shows the different levels of these five lectins in the 210 patients with small GGNs less than 10 mm in diameter. Compared with the noninvasive group, the expressions of terminal α2,6-linked sialic acid (recognized by SNA), high mannose (recognized by NPA and GNA), LacNAc (recognized by DSA), and (GlcNAc)n (recognized by WGA) were decreased in invasive patients.

### 2.4. Validation of Glycosylation Changes of IgG by Lectin Blot

**Figure 3:**
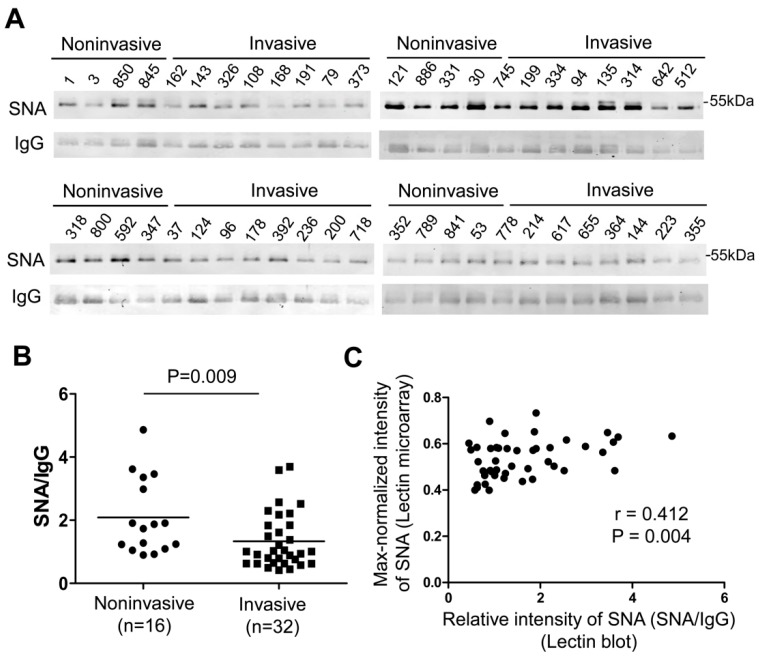
Validation of differential signals of SNA between noninvasive and invasive GGNs by lectin blot. (**A**) SNA-blot of plasma IgG were performed in 16 noninvasive (2 benign and 14 AIS) and 32 invasive (16 MIA and 16 IA) GGN patients. The intensity of IgG detected by Western blot was used as an internal control. (**B**) The expression of SNA signal was normalized to IgG. The ratio (SNA/IgG) between noninvasive and invasive GGNs showed a significant difference (*p* = 0.009). (**C**) Correlation analysis between the intensity of SNA signal from lectin blot and lectin microarray was performed (Spearman correlation coefficient = 0.412, *p* = 0.004). GGN, ground glass nodule; SNA, *Sambucus nigra* agglutinin.

To validate the results of the lectin microarray, we used lectin blotting to detect the signal intensity of SNA on plasma IgG from 16 noninvasive GGNs (2 benign and 14 AIS) and 32 invasive GGNs (16 MIA and 16 IA). The intensity ratios (SNA/IgG) in invasive GGNs was significantly lower than that in noninvasive GGNs (*p* = 0.009) (Figure 3), which was in concordance with the result of the lectin microarray. In addition, correlation analysis revealed that the signal intensities of SNA detected by lectin blot and lectin microarray were strongly correlated (Spearman correlation coefficient = 0.412, *p* = 0.004) (Figure 3C). These results confirmed the reliability of the results of the lectin microarray analysis.

### 2.5. Evaluation of Multilectin Parameters to Assist CT Examination for the Detection of Invasive GGNs

**Figure 4:**
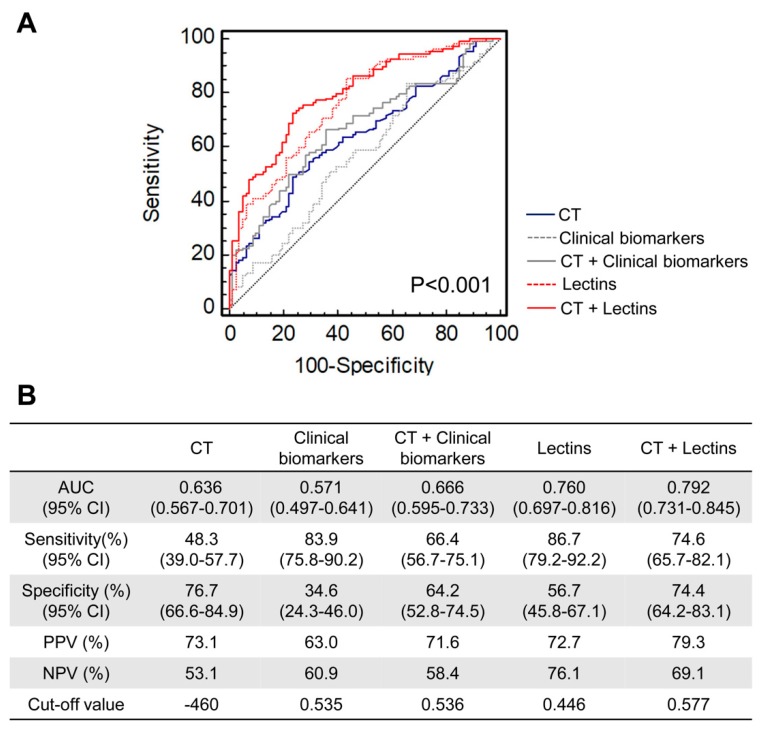
Diagnostic accuracy for prediction of invasive GGNs in the test set. (**A**) Receiver-operating characteristic (ROC) curves for differentiating invasive GGNs from noninvasive GGNs by measuring the CT values, five tumor biomarkers CEA, Cyfra21-1, SCC-Ag, NSE, and CA125 (named “Clinical biomarkers”), five significantly differential lectin signals SNA, DSA, GNA, EEL, and VVA (named “Lectins”), and the combined models with CT values (named “CT + Clinical biomarkers” and “CT + Lectins”, respectively). The area under the ROC curve (AUC) of the indicators was compared by the Delong test (CT vs. CT + Lectins, *p* = 0.007). (**B**) Diagnostic performance of the CT value and the new indicators for the assessment of invasive GGNs. GGN, ground glass nodule; CEA, carcinoembryonic antigen; Cyfra21-1, cytokeratin 19 fragments; SCC-Ag, squamous cell carcinoma antigen; NSE, neuron-specific enolase; CA125, carbohydrate antigen 125; SNA, *Sambucus nigra* agglutinin; DSA, *Datura stramonium* agglutinin; GNA, *Galanthus nivalis* agglutinin; EEL, *Euonymus europaeus* lectin; VVA, *Vicia villosa* agglutinin.

Furthermore, to evaluate the capability of the differential lectin probes as potential biomarkers to assist CT examination for the detection of invasive patients with small GGNs less than 10 mm in diameter, receiver-operating characteristic (ROC) analysis was used. Based on stepwise multiple logistic regression analysis using eight differential lectins (Table 2), SNA, DSA, GNA, EEL, and VVA were identified as the independent variables most closely associated with susceptibility of GGN invasiveness using a logistic forward selection (likelihood ratio) algorithm (*p* < 0.01). Furthermore, we constructed new diagnosis models using these five lectins alone (named “Lectins”), as well as these five lectins combined with the CT value (named “CT + Lectins”) in a binary logistic regression analysis. In addition, to compare the routine serological indicators, we also constructed diagnosis models using five routine tumor biomarkers, CEA, Cyfra21-1, SCC-Ag, NSE, and CA125 (named “Clinical biomarkers”), as well as these five biomarkers combined with the CT value (named “CT + Clinical biomarkers”). As shown in Figure 4, the diagnostic ability of current clinical biomarkers for invasiveness in GGN was poor; even the combination model “CT + Clinical biomarkers” was just comparable to that of CT values. Although the sensitivity (66.4%) of diagnosing invasive GGNs was improved, the specificity (64.2%) was decreased. By contrast, the model “Lectins” showed a better diagnostic performance than CT values with an improved area under the curve (AUC) (from 0.636 to 0.760, *p* = 0.01). Especially, the diagnostic ability of the combined model “CT + Lectins” showed a remarkable improvement compared with CT values alone. The AUC (95% CI) to distinguish between noninvasive and invasive GGNs (0.792, 0.731–0.845) was superior to CT values (0.636, 0.567–0.701) (*p* < 0.001), with a sensitivity of 74.6% and specificity of 74.4%. These results indicate that the specific glycosylation changes of plasma IgG as a serological indicator might assist current CT examination to improve the diagnosis accuracy of invasive GGNs, which is better than current tumor biomarkers.

## 3. Discussion

In China, the incidence of pulmonary nodules, including GGNs, has dramatically increased in the last few years. According to undisclosed data of Shanghai Chest Hospital, nearly 3000 patients with preoperative CT manifestations of GGN underwent surgical resection one year. However, 20% of these patients were finally shown to be noninvasive lesions by postoperative pathological diagnosis. Currently, LDCT screening is recommended as an efficient method for the early detection of lung cancer in China [25], with a high sensitivity to detect small pulmonary nodules, even those less than 10 mm in diameter. However, because there is considerable overlap among the imaging features of AAH, AIS, and IA, it is challenging to differentiate noninvasive from invasive nodules by a visual assessment of morphological characteristics based on CT imaging [8]. For example, the sensitivity of CT values for detecting invasive GGNs in our study was only 48.3%. Therefore, the development of a novel serological test that can assist CT scans in improving the discrimination accuracy between noninvasive and invasive GGNs before surgery is of great importance. 

Glycosylation is a highly heterogeneous post-translation modification, which shows different isoforms under different pathological states. In this study, we used a lectin microarray-based strategy to analyze the glycosylation changes in plasma IgG and identify potential glycobiomarker for invasive GGNs. Although it yields less detailed structural information compared with traditional approaches using mass spectrometry, the lectin microarray is a simple and highly sensitive method to directly obtain global glycosylation profiling without the need for releasing glycans [26]. Such features make it suitable for the initial detection of glycosylation changes in various biological samples. Once we have found and validated one or more lectin probes with biological significance, they can be easily used as potential disease biomarkers in routine tests for a large number of clinical specimens by lectin-antibody ELISA. Currently, this approach has proved useful in screening novel biomarkers for disease diagnosis, including lung cancer [27]. 

We found several glycosylation features of IgG had significantly changed during the progression of GGN using the lectin microarray-based strategy. First, a significant decrease in α2,6-linked sialylation (recognized by SNA) was observed in invasive GGNs, which is consistent with a previous study reporting that monosialyl IgG oligosaccharides were significantly lower in lung cancer patients than in healthy controls using fluorophore-assisted carbohydrate electrophoresis (FACE) [28] or LC-MS [23]. The decreased level of α2,6-linked sialylation was also observed in many other diseases, such as arthritis [29], and was associated with a poorer prognosis in colorectal cancer [30]. The biological function of sialylation on IgG has been recognized as a switch between pro- and anti-inflammatory effects. It was reported that α2,6-linked sialylation on IgG has anti-inflammatory functions based on its ability to increase the activation threshold of innate effector cells to immune complexes [31] or impair their efficacy to induce complement-mediated cytotoxicity (CDC) [32]. Thus, our result showing the decreased sialylation on IgG suggests a proinflammatory characteristic in invasive GGN patients. Another important finding in our study was the marked decrease of high mannose (recognized by GNA, NPA, and HHL) in invasive GGNs. This result is consistent with previous reports showing that the relative abundances of high mannose-type glycans were significantly lowered in the serum of NSCLC [33], but in contrast with the report of lung adenocarcinoma tissue [34]. IgG is secreted by B-cells and the glycosylation of IgG is mainly regulated by B-cell-specific glycosyltransferase or other exogenous posttranslational modification within the bloodstream [35]. Therefore, it is unsurprising to note the opposite changes of IgG glycans in the plasma compared with tissues. In addition, IgG containing Fc high-mannose glycans have a more rapid clearance rate than other glycoforms because binding to the mannose receptor facilitates the uptake of IgG complexes by macrophages and dendritic cells [36]. Taken together, we assume that plasma IgG in invasive GGN patients is proinflammatory with a long half-life.

Of note, we also observed a significantly decreased signal of DSA, which mainly recognizes Galβ1-3GlcNAc (LacNAc) on tri- and tetra-antennary structures. There have been few reports about tri- and tetra-antennary structures on human IgG. However, with the development of sensitive detection technologies, increasing numbers of trace glycans, including the tri- and tetra-antennary structures, have been observed on serum IgG, some of which have been identified as novel biomarkers for disease [37]. Our study findings suggest the high sensitivity of the lectin microarray will be useful for the discovery of trace glycans. To date, it is unclear whether tri- and tetra-antennary structures represent any new functional benefit; therefore, a functional study of these trace glycans would be of great interest in the future. 

In addition, in the field of the IgG glycome, the molecular mechanisms between altered secreted IgG glycans and disease progression has long been an important but complicated issue. Although it is still not fully elucidated, the IgG glycome might be a host defense reaction response to the presence of tumors [38], which are reliant on glycosyltransferases and glycosidases mainly present in B and plasma cells [39]. Thus, multiples factors, such as (1) genetic variants in glycosyltransferases [40,41], (2) epigenetic alterations [42], and (3) the cellular environment including cytokines [43,44,45], which might influence the expression or activity of related enzymes may modulate the glycosylation of IgG [46,47,48]. It is generally considered that the development of lung adenocarcinoma follows a linear multistep progression, from noninvasive lesions (AAH and AIS) to MIA, followed by IA [49,50]. Despite the reports of numerous intrinsic factors, such as mutations of EGFR and KRAS, in cancer cells contributing to the transition from noninvasive to invasive lesions [51,52], inflammatory cells and cytokines in the lung microenvironment also play a crucial role in the malignant progression [53,54], and which also influence the expression or activity of glycotransferase [55,56]. Among these, the percentage of T helper 17 (Th17) cells and related cytokine interleukin 17 (IL-17) were increased in patients with extensive tumor invasion [57,58,59]. In addition, another cytokine IL-22, produced by Th17 cells, enhanced the migration and invasion in NSCLC cell lines [60]. These results suggest that Th17 cells may be significantly correlated with the invasive potential of lung adenocarcinoma. With regard to sialylation, a recent study showed that IL-23-activated Th17 cells suppressed the β-galactoside α2,6-sialyltransferase 1 (ST6Gal1) expression in developing plasmablasts and plasma cells in an IL-21- and IL-22-dependent manner, which resulted in a decrease in serum IgG sialylation [45]. Therefore, we speculate that changes in the level of cytokines in pulmonary-infiltrating cells leads to the change of glycosyltransferases in plasma cells, which may be one of the mechanisms that results in the change of IgG glycosylation, such as the decreased sialyation in invasive GGNs. Moreover, it has been reported that IgG sialylation can also occur in the bloodstream environment by extracellular ST6GAL1 independent of the B cell secretory pathway [35,61]. Therefore, we preliminarily detected the level of plasma ST6GAL1 in 80 GGN patients (noninvasive group, *n* = 40; invasive group, *n* = 40) by ELISA. Although the plasma level of ST6GAL1 was relatively low with an abundance of 100 to 150 pg/mL, in a majority of these patients, there was a decreased trend for plasma ST6GAL1 levels in invasive GGNs (Supplementary Figure S5), which might be one of the reasons causing the decrease in IgG sialylation in invasive GGNs.

In this study, we provided a multilectin assay that can evaluate different glycosylation changes in IgG simultaneously. This assay can assist CT scans in discriminating between noninvasive and invasive GGNs with a higher diagnostic accuracy (AUC: 0.792, sensitivity: 74.6%, and specificity: 74.4%) compared with current clinical biomarkers, and provide surgeons with complementary information about the pathological changes of GGN. To date, increasing studies have paid attention to the genetic and transcriptomic features of noninvasive lesions [10,62]; however, no serological evidence is available to suggest which kind of GGN should be resected. Our results suggest that an early glycosylation feature of IgG might predict the invasiveness potential with a better performance than current tumor biomarkers in assisting clinical decision making. More importantly, using IgG as an example, we suggest that lectin-based glycomics may provide a new method for future liquid biopsies, which may be reliable enough to be commonly used in pre-screening and early cancer diagnostics for some redoubted disease.

The present study still had some limitations. First, although we used two relatively independent cohorts for the identification of significant lectin probes, the data in this study should be confirmed using more samples in a multi-center study. In addition, the advantage of the lectin microarray is the ability to rapidly and efficiently select potential disease-specific lectin probes for further clinical analysis. However, because some lectins have cross reactivity with different glycans, a more complex interpretation method considering the signals of lectins with similar or different binding specificities needs to be developed in the future. Moreover, several reports have pointed out more or less lot-to-lot variations between lectin chips, which may be caused by variations during the purification of lectins, the production of the epoxy-coated glass, or the spotting process [63]. Thus, future technical improvement will make it possible to realize the absolute quantification of lectin detection without taking care of the lot-to-lot variation.

## 4. Materials and Methods 

### 4.1. Patients and Study Design

From January 2015 to January 2017, 1715 patients who underwent preoperative chest CT examination in Shanghai Chest Hospital were enrolled. Of these, 689 patients were diagnosed as pure GGN or mixed GGN on CT images. Four hundred and thirty-six patients remained in this study based on the inclusion criteria: (1) Underwent complete surgical resection, (2) no concurrent solid pulmonary nodules, (3) maximum lesion size ≤ 15 mm, and (4) obtained a definitive pathological diagnosis. 

All resected specimens were formalin fixed and stained with hematoxylin–eosin for pathological diagnosis in the department of pathology in Shanghai Chest Hospital. Each specimen was histopathological analyzed by two experienced thoracic pathologists independently according to the new lung adenocarcinoma classification [4]. The discordant cases were subsequently discussed in a consensus meeting until a consensus was obtained. When considering treatments for GGN, AIS can be closely followed up without urgent surgical resection because of its slow growth (although it is an *in situ* adenocarcinoma) to exclude the possibility of AAH or other benign lesions. By contrast, MIA needs prompt surgical excision to prevent the development of invasive adenocarcinoma and distant metastasis. The purpose of this study was to identify complementary indicators for surgical intervention. Therefore, we divided patients into a noninvasive group including benign cases (inflammation, hemorrhage, and fibrosis), AAH, and AIS, and an invasive group including MIA and IA. 

A venous blood sample was obtained preoperatively from each patient during the morning fasting state. The plasma samples were aliquoted and stored at −80 °C until analysis. In total, 302 patients were finally used for lectin microarray analysis, of which 92 specimens collected between January 2015 and September 2015 were considered as the discovery set and the other 210 specimens ≤ 10 mm in diameter were used as the test set. The discovery set was used for a preliminary screen of potential glycosylation features related to GGN invasiveness, and the test set was used to subsequently evaluate these potential candidates to find specific differential glycosylation features in small GGNs less than 10 mm in diameter. Sample preparation and analysis of the two sample sets were performed independently with a 1-year interval. This study was conducted in accordance with the principles of the Declaration of Helsinki and approved by the Ethical Committee of Shanghai Chest Hospital. Written informed consent was obtained from all patients.

### 4.2. CT Examination and Biochemical Data

CT imaging features were retrospectively analyzed by two thoracic radiologists who were blinded to pathological diagnosis, and a consensus was reached. The patients’ age and sex, maximal diameter, and maximum CT attenuation value for each nodule were recorded. If multiple GGNs were found in one specimen, the characteristics of the largest nodule were recorded.

In addition, the following routine tumor biomarkers were measured: Carcinoembryonic antigen (CEA), cytokeratin 19 fragments (Cyfra21-1), squamous cell carcinoma antigen (SCC-Ag), neuron-specific enolase (NSE), and carbohydrate antigen 125 (CA125).

### 4.3. IgG Isolation from Plasma

IgG for lectin microarray was purified from plasma using Protein G Magnetic Beads (Bio-Rad Laboratories) following the manufacturer’s instructions with some modifications in accordance with Kuno et al. [64]. Briefly, the plasma samples were diluted 1:10 with phosphate-buffered saline (PBS) containing 0.2% SDS and then heated at 95 °C for 10 min. The resulting solution (5 μL) of 10-fold-diluted plasma was incubated with 10 μL Protein G beads and 85 μL PBSTx (0.5% TritonX-100 in PBS) at 4 °C for 5 min while shaking at 1400 rpm continuously. Subsequently, unbound protein was removed by washing with five column volumes of PBSTx. Bound IgG was eluted with 20 μL of PBS containing 0.2% SDS by heat denaturation at 95 °C for 5 min, and then placed immediately on ice for 1 min. Each 1 μL of IgG elution was used for purity detection by sliver staining, and the other elution fractions were stored at −80 °C until further use.

### 4.4. Lectin Microarray Analysis

Lectin microarray analysis was basically performed as described previously [63]. Briefly, 5 μL of IgG elution was fluorescence-labeled with 10 μg of Cy3-succinimidyl ester (GE Healthcare) at room temperature (RT) for 1 h in the dark. The sample solution was adjusted to 100 μL with probing buffer (500 mM glycine, 1 mM CaCl_2_, and 1 mM MnCl_2_ in Tris-buffered saline containing 1.0% Triton X-100) and incubated at RT for 2 h to block the excess fluorescent reagent. Each 2.5-μL aliquot of Cy3-labeled elution was applied to one well on a lectin microarray chip (LecChip™; GlycoTechnica) containing triplicate spots of 45 lectins (Supplementary Table S1). After incubation for 16 h at 20 °C, the chip was scanned using an evanescent-field fluorescence scanner (GlycoStation™ Reader 1200; GlycoTechnica) at Gain 75, 85, 95, 105, 115, and 125, and the data were analyzed with GlycoStation™ ToolsPro 1.5 (GlycoTechnica). The net intensity was calculated by subtracting the background from the signal intensity. We selected the scanning data under appropriate gain conditions, which provided the net intensities of all positive spots < 40,000. The relative intensity of each lectin was normalized by the strongest signal intensity of all lectins (“max-normalization” method) according to the principles described previously [24]. 

### 4.5. Lectin Blot and Western Blot Analysis

These analysis was basically performed as described previously [65,66]. Five microliters of IgG elution were separated by 10% sodium dodecyl sulphate–polyacrylamide gel electrophoresis (SDS–PAGE) and transferred to cellulose acetate membranes (GE Healthcare). The membrane was blocked with PBS containing 5% *w*/*v* bovine serum albumin (BSA) at RT for 2 h prior to incubation with biotin-labeled SNA (1:1000; Vector Laboratories) and goat anti-human IgG antibody (1:5000; Merck) at 4 °C overnight. After washing with PBST (0.1% Tween-20 in PBS), the membrane was incubated with Streptavidin-680 (1:5000; Li-COR) and Alexa Fluor^®^ 800 anti-goat IgG secondary antibody (1:5000; Life Technologies). The signals were visualized by an Odyssey Infrared Imaging System (Li-COR) and quantified by Quantity One (Bio-Rad). The intensity ratios (SNA/IgG) were calculated.

### 4.6. Statistical Analysis

Statistical calculations were conducted with SPSS version 16.0 and GraphPad Prism 5. Categorical data were analyzed using the chi-square test and continuous variables were compared with the Mann–Whitney U test or student’s t-test. Linear relation analysis was assessed using Spearman rank correlation or Pearson correlation coefficient. The diagnostic models were constructed according to the CT values, glycopattern of IgG, or other clinical biomarkers based on a logistic regression analysis. A forward selection (likelihood ratio) algorithm was used to select variables to include in the logistic regression model. In addition to evaluating the diagnostic performance of different diagnostic models in predicting the pathological invasiveness in GGN, receiver-operating characteristic (ROC) curve analysis was performed. Areas under the ROC curves were compared by the Delong test. Optimal cut-off values were obtained from Youden’s index. A *p* value < 0.05 in all cases was considered statistically significant.

## 5. Conclusions

In conclusion, this study provided a new attempt to identify serological biomarkers to predict pathological changes in the very early stages of lung cancer. We described, for the first time, changes in the glycosylation pattern of plasma IgG correlated with the pathological states of GGNs using a simple lectin microarray-based strategy. Our results suggest that a multilectin assay based on plasma IgG glycosylation might be used as in vitro complementary test to enhance preoperative determination of the invasiveness of small-sized GGNs, and help clinicians to adopt proper clinical management to avoid overtreatment of noninvasive patients.

## Figures and Tables

**Table 1:** Clinical characteristics of the patients in this cohort.

	Discovery Set (*n* = 92)			Test Set (*n* = 210)		
	Noninvasive Group (*n* = 37)	Invasive Group (*n* = 55)	*p* ^3^	Noninvasive Group (*n* = 90)	Invasive Group (*n* = 120)	*p* ^3^
Age (year) ^1^	51.19 ± 10.98	54.69 ± 9.72	0.176	51.50 ± 10.77	52.08 ± 11.19	0.639
Gender (male/female)	8/29	12/43	0.982	19/71	29/91	0.602
Nodule diameter (mm) ^1^	8.7 ± 2.5	9.3 ± 2.4	0.240	7.7 ± 1.4	8.2 ± 1.5	0.009
Pathology ^2^			/			/
Benign	4 (10.8%)	/		10 (4.8%)	/	
AAH	5 (13.5%)	/		4 (1.9%)	/	
AIS	28 (75.7%)	/		76 (36.2%)	/	
MIA	/	34 (61.8%)		/	84 (40.0%)	
IA	/	21 (38.2%)		/	36 (17.1%)	
Clinical parameters ^1^						
CEA (ng/mL)	1.55 ± 0.60	1.95 ± 0.94	0.059	1.39 ± 0.90	1.67 ± 1.31	0.099
Cyfra21-1 (ng/mL)	1.44 ± 0.67	1.48 ± 0.99	0.647	1.39± 0.71	1.34 ± 0.64	0.793
SCC-Ag (ng/mL)	0.85 ± 0.43	0.80 ± 0.34	0.978	1.20 ± 3.16	0.92 ± 0.93	0.660
NSE (ng/mL)	11.64 ± 4.46	11.38 ± 3.27	0.845	13.87 ± 5.05	13.55 ± 5.12	0.671
CA125 (ng/mL)	16.19 ± 16.39	11.57 ± 5.71	0.454	12.67 ± 11.57	11.73 ± 6.70	0.638
CT value (HU)	−542.28 ± 132.68	−433.24 ± 179.68	0.007	−515.50 ± 207.82	−421.25 ± 202.35	0.001

^1.^ Data are presented as mean ± SD unless indicated otherwise. ^2^ Data are presented as the numbers with percentage in parentheses. ^3^ *p* values are derived from the Pearson chi-square test or Mann–Whitney U test between noninvasive lesions and invasive lesions. AAH, atypical adenomatous hyperplasia; AIS, adenocarcinoma *in situ*; MIA, minimally invasive adenocarcinoma; IA, invasive adenocarcinoma; Benign cases include inflammation, hemorrhage, and fibrosis.

**Table 2:** Significant lectin signals between the noninvasive and invasive groups validated in the test set.

	Binding Specificity	Test Set ^1^			
		Noninvasive Group (*n* = 90)	Invasive Group (*n* = 120)	*p* ^2^	
SNA	Siaα2-6Gal/GalNAc	13.35 ± 2.86	12.50 ± 2.37	0.046	<0.001
DSA	(GlcNAc)n, polyLacNAc and branched LacNAc	51.33 ± 8.20	48.78 ± 9.32	0.046	0.001
NPA	Non-substituted α1-6Man	21.85 ± 3.44	20.55 ± 3.37	0.009	
GNA	Non-substituted α1-6Man	6.08 ± 1.80	5.57 ± 1.52	0.045	0.001
HHL	Non-substituted α1-6Man	0.66 ± 0.22	0.60 ± 0.18	0.025	
EEL	Galα1-3(Fuc α1-2)Gal	0.15 ± 0.06	0.12 ± 0.05	0.002	<0.001
VVA	terminal GalNAc	0.03 ± 0.04	0.04 ± 0.03	0.009	0.001
WGA	(GlcNAc)n and multivalent Sia	23.88 ± 5.72	21.27 ± 5.62	0.001	

^1^ Relative intensity are presented as the mean ± SD unless indicated otherwise. ^2^ *p* values are derived from the Mann–Whitney U test for univariate analysis or stepwise multiple logistical regression for multivariate analysis. SNA, *Sambucus nigra* agglutinin; DSA, *Datura stramonium* agglutinin; NPA, *Narcissus pseudonarcissus* agglutinin; GNA, *Galanthus nivalis* agglutinin; HHL, *Hippeastrum hybrid* lectin; EEL, *Euonymus europaeus* lectin; VVA, *Vicia villosa* agglutinin; WGA, *Weat germ* agglutinin.

## Supplementary Materials

The following are available online at https://www.mdpi.com/1420-3049/25/1/28/s1, Figure S1: Scheme of the lectin microarray-based strategy to discover potential glycobiomarkers of plasma IgG for invasive GGNs. IgG, immunoglobin G; GGN, ground glass nodule, Figure S2. Sliver staining of purified IgG from plasma. (A) IgG from crude plasma were extracted using commercial Protein G magnetic beads incubating 5 min, 30 min and 1 h, respectively. The elution fraction and flow through (FT) fraction were separated by SDS-PAGE and visualized by silver staining. Each sample had two duplications (① and ②). Different quantities of bovine serum albumin (BSA) were used as controls. (B) Representative silver staining results of plasma IgG elution from GGNs were shown. IgG, immunoglobin G; GGN, ground glass nodule, Figure S3: Optimization the concentration ranges of IgG elution for lectin microarrays. (A) A serial dilution of Cy3-labeled IgG elution (10, 5, 2.5, 1.25, 0.625, and 0.313 μL) from a healthy volunteer were subjected to the lectin microarray analysis. Negative control (NC) was using PBS containing 1% TritonX-100 instead of IgG elution. (B) The intensities of 45 lectin signals under different gain conditions (Gain75, 85, 95, 105, 115, and 125) were shown. The dynamic range with sufficient linearity of fluorescence intensities in lectin microarray (from 1000 to 40,000) were shown in red dashed line. IgG, immunoglobin G, Figure S4: Reproducibility of the lectin microarray analysis. 2.5 μL aliquots of IgG elution from one GGN patient (#393) were subjected to six LecChips from different bags of array. The mean, SD, and CV values of intensities for all the positive lectins were calculated. IgG, immunoglobin G; GGN, ground glass nodule, Figure S5: Detection of the plasma level of β-galactoside α2,6-sialyltransferase 1 (ST6Gal1) in 80 GGN patients by ELISA. The P value was calculated by two-tail student’s t-test. GGN, ground glass nodule, Table S1: Binding specificities of 45 lectins on LecChip, Table S2: The Pearson correlation coefficients of the 45 lectin signals among the six LecChips, Table S3: Significant lectin signals between the noninvasive and invasive groups in discovery set.
