# Supplementary material for: Glycomic Signatures of Plasma IgG Improve Preoperative Prediction of the Invasiveness of Small Lung Nodules
Source: Molecules. 2019 Dec 20;25(1):28. doi: 10.3390/molecules25010028 (PMC6982969; doi:10.3390/molecules25010028)
Supplement: Supplementary file 1 [file molecules-25-00028-s001.zip › molecules-638357-proof-supplementary-layout/molecules-638357-proof-Supplementary Figures-layout.docx]

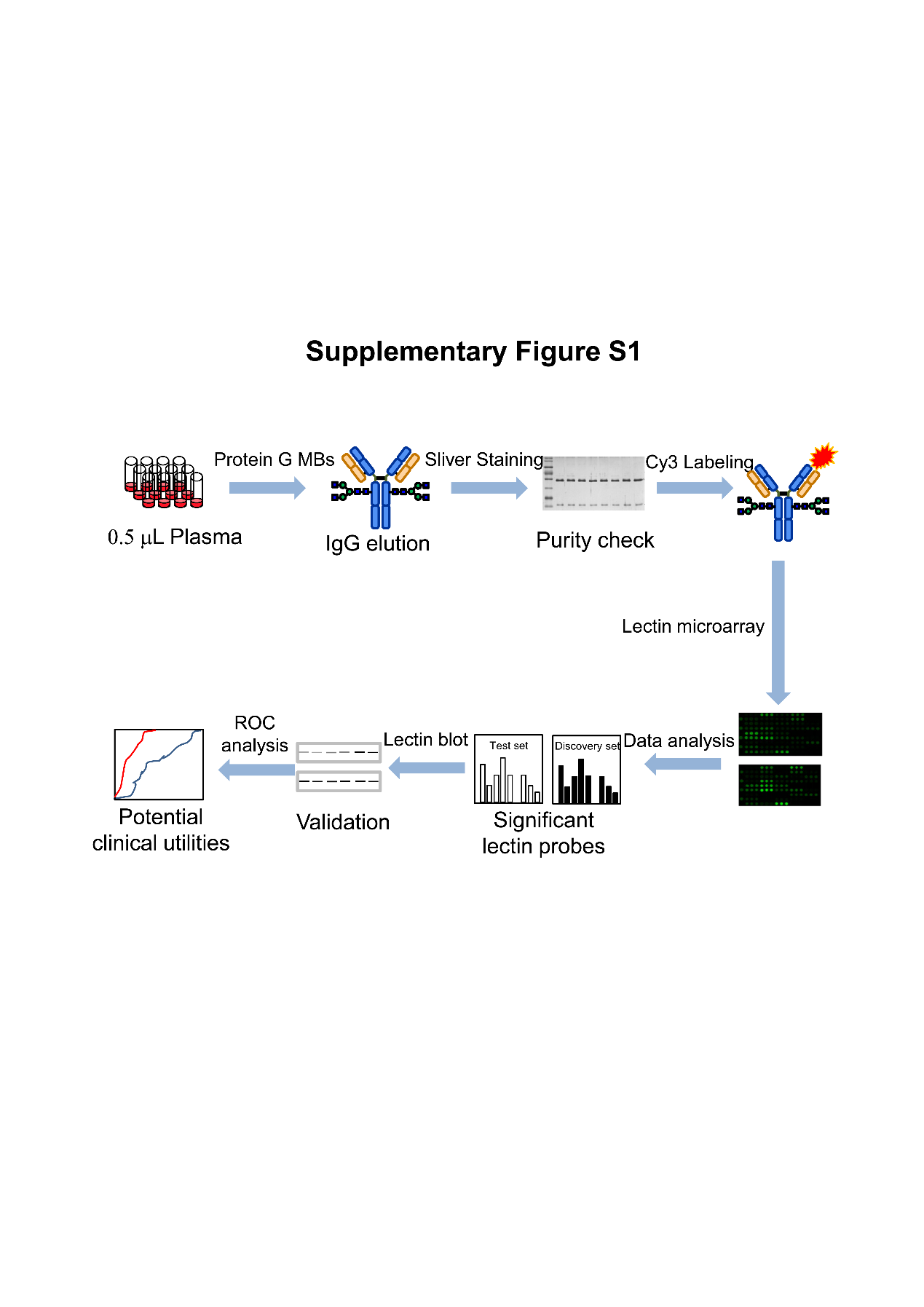


**Supplementary Figure S1.** Scheme of the lectin microarray-based strategy to discover potential glycobiomarkers of plasma IgG for invasive GGNs. IgG, immunoglobin G; GGN, ground glass nodule.


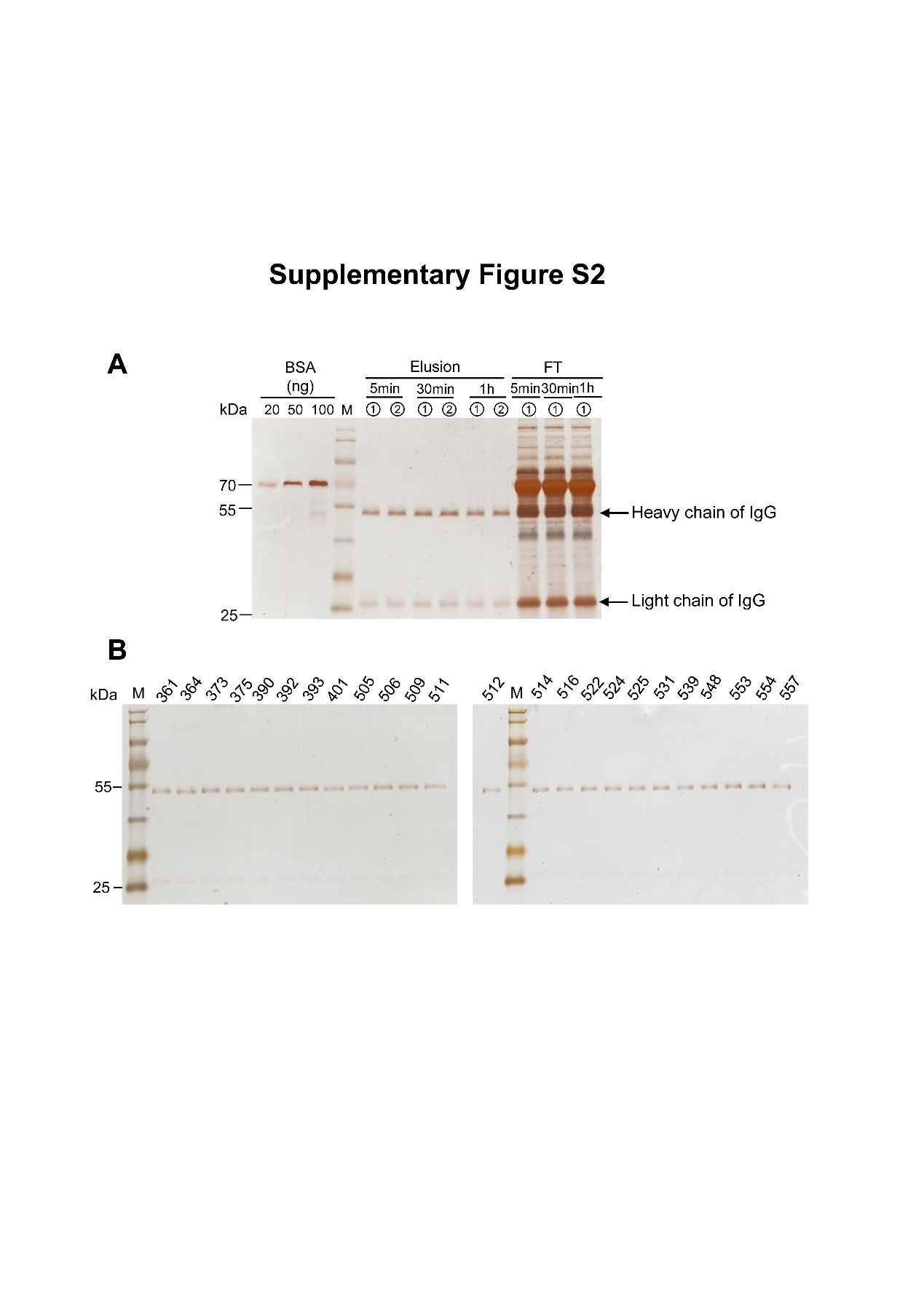


**Supplementary Figure S2.** Sliver staining of purified IgG from plasma. (**A**) IgG from crude plasma were extracted using commercial Protein G magnetic beads incubating 5 min, 30 min and 1 h, respectively. The elution fraction and flow through (FT) fraction were separated by SDS-PAGE and visualized by silver staining. Each sample had two duplications (① and ②). Different quantities of bovine serum albumin (BSA) were used as controls. (**B**) Representative silver staining results of plasma IgG elution from GGNs were shown. IgG, immunoglobin G; GGN, ground glass nodule.


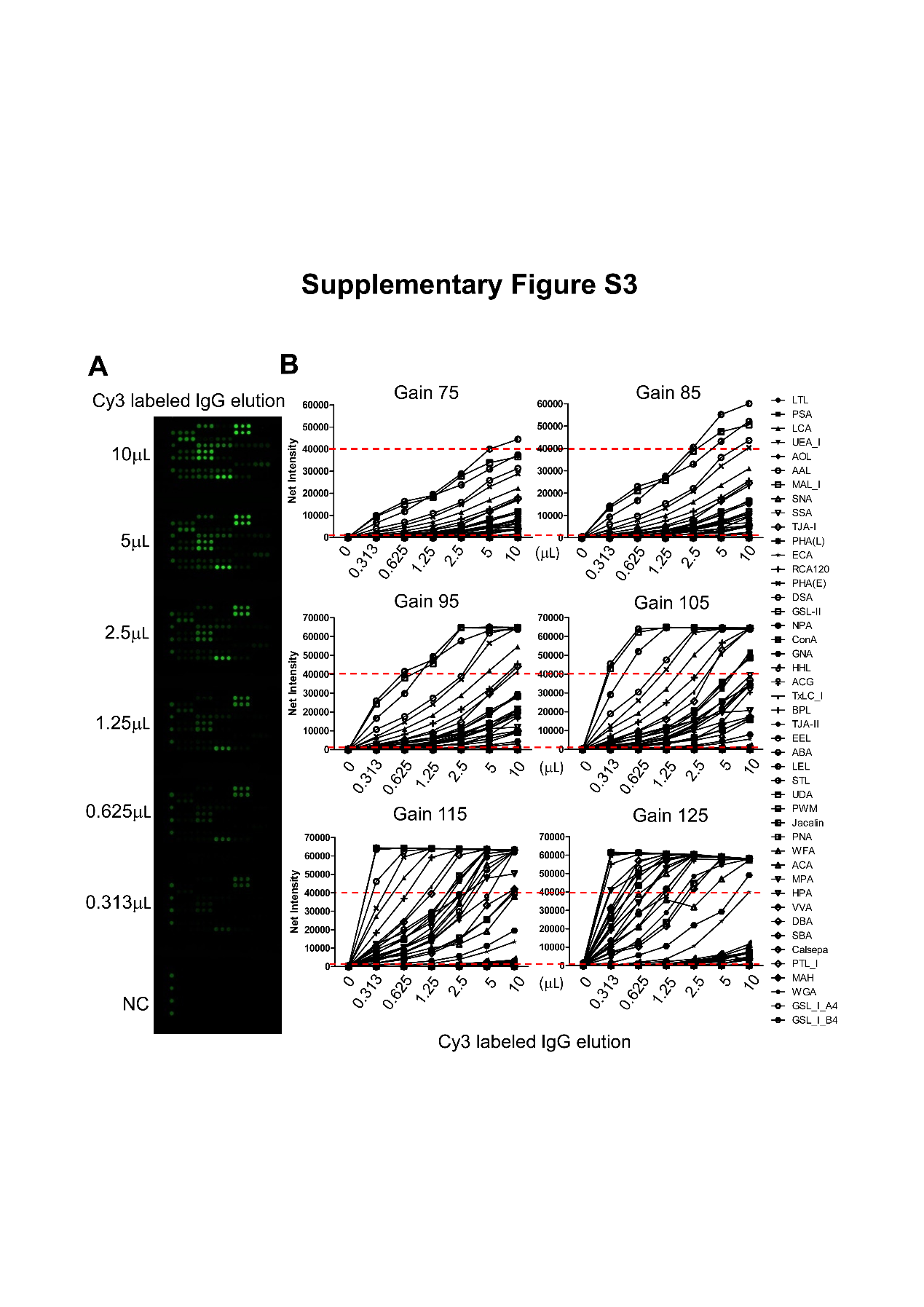


**Supplementary Figure S3.** Optimization the concentration ranges of IgG elution for lectin microarrays. (**A**) A serial dilution of Cy3-labeled IgG elution (10, 5, 2.5, 1.25, 0.625, and 0.313 μL) from a healthy volunteer were subjected to the lectin microarray analysis. Negative control (NC) was using PBS containing 1% TritonX-100 instead of IgG elution. (**B**) The intensities of 45 lectin signals under different gain conditions (Gain75, 85, 95, 105, 115, and 125) were shown. The dynamic range with sufficient linearity of fluorescence intensities in lectin microarray (from 1000 to 40,000) were shown in red dashed line. IgG, immunoglobin G.


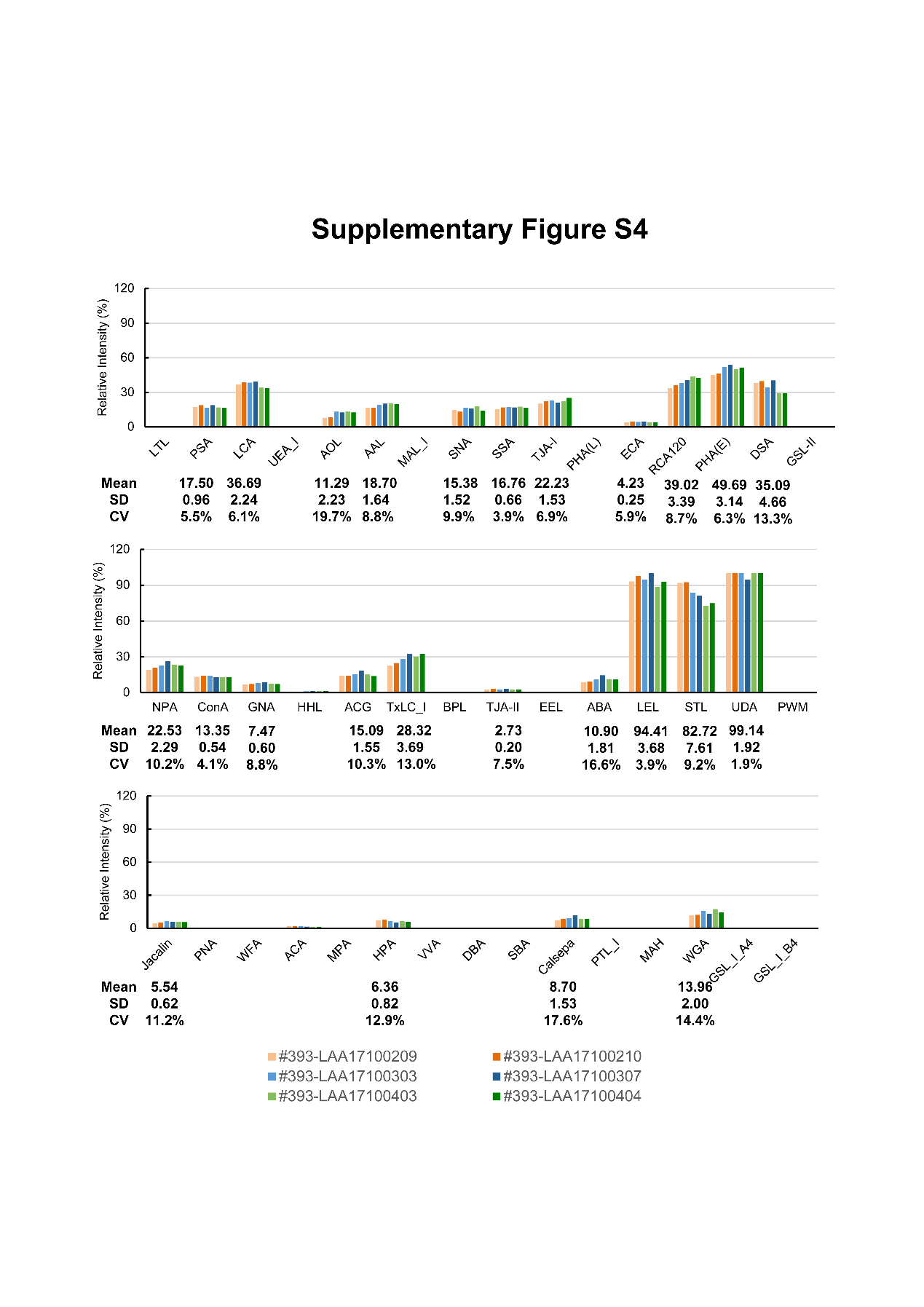


**Supplementary Figure S4.** Reproducibility of the lectin microarray analysis. 2.5 μL aliquots of IgG elution from one GGN patient (#393) were subjected to six LecChips from different bags of array. The mean, SD, and CV values of intensities for all the positive lectins were calculated. IgG, immunoglobin G; GGN, ground glass nodule.


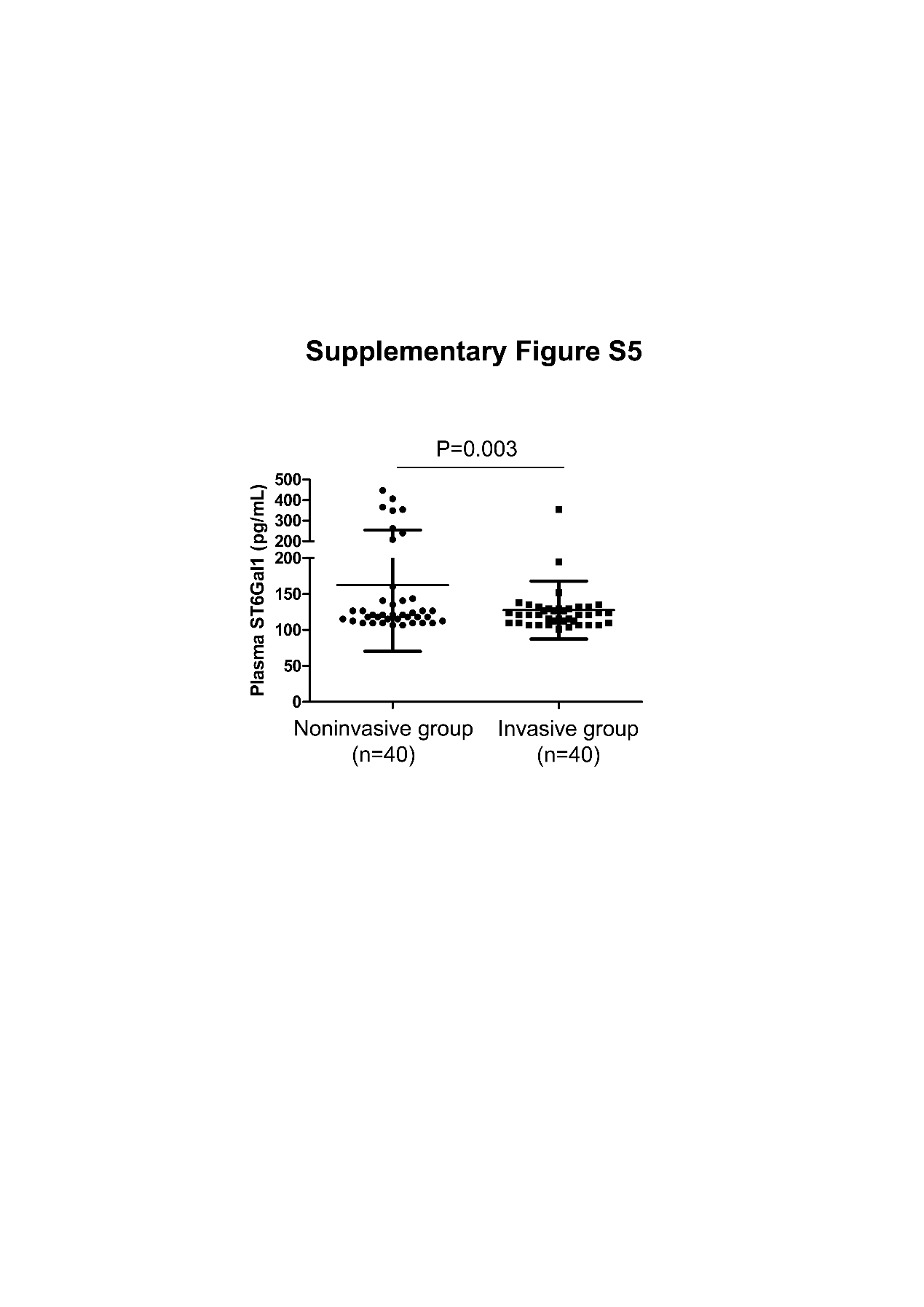


**Supplementary Figure S5.** Detection of the plasma level of β-galactoside α2,6-sialyltransferase 1 (ST6Gal1) in 80 GGN patients by ELISA. The P value was calculated by two-tail student’s t-test. GGN, ground glass nodule.
