# Supplementary material for: Glycomic Signatures of Plasma IgG Improve Preoperative Prediction of the Invasiveness of Small Lung Nodules
Source: Molecules. 2019 Dec 20;25(1):28. doi: 10.3390/molecules25010028 (PMC6982969; doi:10.3390/molecules25010028)
Supplement: Supplementary file 1 [file molecules-25-00028-s001.zip › molecules-638357-proof-supplementary-layout/molecules-638357-proof-Supplementary Table-layout.docx]

**Supplementary Table S1.** Binding specificities of 45 lectins on LecChip.

| **No.** | **Lectins** | **Binding Specificity** | **Origin** |
| --- | --- | --- | --- |
| 1 | LTL | Fucα1-3GlcNAc, Sia-Le^x^ and Le^x^ | *Lotus tetragonolobus* |
| 2 | PSA | Fucα1-6GlcNAc and α-Man | *Pisum sativum* |
| 3 | LCA | Fucα1-6GlcNAc, α-Man and α-Glc | *Lens culinaris* |
| 4 | UEA-I | Fucα1-2(Galβ1-4)GlcNAc | *Ulex europaeus* |
| 5 | AOL | Fucα1-6GlcNAc and  Fucα1-2(Gal β1-4)GlcNAc | *Aspergillus oryzae* |
| 6 | AAL | Fucα1-6GlcNAc and Le^x^ | *Aleuria aurantia* |
| 7 | MAL | Siaα2-3Galβ1-4GlcNAc | *Maackia amurensis* |
| 8 | SNA | Siaα2-6Gal/GalNAc | *Sambucus nigra* |
| 9 | SSA | Siaα2-6Gal/GalNAc | *Sambucus sieboldiana* |
| 10 | TJA-I | Siaα2-6Gal/GalNAc and Gal | *Trichosanthes japonica* |
| 11 | PHA-L | Tri- and tetra-antennary complex-type *N*-glycans | *Phaseolus vulgaris* |
| 12 | ECA | Galβ1-4GlcNAc | *Erythrina cristagalli* |
| 13 | RCA120 | Galβ1-4GlcNAc | *Ricinus communis* |
| 14 | PHA-E | Bisecting GlcNAc and biantennary *N*-glycans | *Phaseolus vulgaris* |
| 15 | DSA | (GlcNAcβ1-4)_n_, polyLacNAc and branched LacNAc | *Datura stramonium* |
| 16 | GSL-II | Agalactosylated *N*-glycan and GlcNAc | *Griffonia simplicifolia* |
| 17 | NPA | non-substituted α1-6Man | *Narcissus pseudonarcissus* |
| 18 | ConA | α-Man (inhibited by presence of bisecting GlcNAc) | *Canavalia ensiformis* |
| 19 | GNA | non-substituted α1-6Man | *Galanthus nivalis* |
| 20 | HHL | non-substituted α1-6Man | *Hippeastrum hybrid* |
| 21 | ACG | Siaα2-3Gal β1-4GlcNAc | *Agrocybe cylindracea* |
| 22 | TxLC-I | Man_3_ core, bi- and tri-antennary complex-type *N*-glycan and GalNAc | *Tulipa gesneriana* |
| 23 | BPL | Galβ1-3GalNAc and GalNAc | *Bauhinia purpurea alba* |
| 24 | TJA-II | β-GalNAc and Fucα1-2Gal | *Trichosanthes japonica* |
| 25 | EEL | Galα1-3(Fuc α1-2)Gal | *Euonymus europaeus* |
| 26 | ABA | Gal, Galβ1-3GalNAc and sialyl-T | *Agaricus bisporus* |
| 27 | LEL | (GlcNAc)_n_ and polyLacNAc | *Lycopersicon esculentum* |
| 28 | STL | (GlcNAc)_n_ and polyLacNAc | *Solanum tuberosum* |
| 29 | UDA | (GlcNAc)_n_ and polyLacNAc | *Urtica dioica* |
| 30 | PWM | (GlcNAc)_n_ and polyLacNAc | *Phytolacca americana* |
| 31 | Jacalin | Galβ1-3GalNAcα-Thr/Ser (T) and GalNAcα-Thr/Ser (Tn) | *Artocarpus integrifolia* |
| 32 | PNA | Galβ1-3GalNAcα-Thr/Ser (T) | *Arachis hypogaea* |
| 33 | WFA | Terminal GalNAc (e.g., GalNAcβ1-4GlcNAc) and Galβ1-3(-6)GalNAc | *Wisteria floribunda* |
| 34 | ACA | Galβ1-3GalNAcα-Thr/Ser (T) | *Amaranthus caudatus* |
| 35 | MPA | Galβ1-3GalNAcα-Thr/Ser (T) and GalNAcα-Thr/Ser (Tn) | *Maclura pomifera* |
| 36 | HPA | α-linked terminal GalNAc | *Helix pomatia* |
| 37 | VVA | GalNAcα-Thr/Ser (Tn) and GalNAcα1-3 Gal | *Vicia villosa* |
| 38 | DBA | GalNAcα-Thr/Ser (Tn) and GalNAcα1-3GalNAc | *Dolichos biflorus* |
| 39 | SBA | Terminal GalNAc (especially GalNAcα1-3Gal) | *Glycine max* |
| 40 | Calsepa | High Man and *N*-glycans including bisecting GalNAc | *Calystegia sepium* |
| 41 | PTL-I | α-GalNAc and Gal | *Psophocarpus tetragonolobus* |
| 42 | MAH | Siaα2-3Gal β1-3(Sia α2-6) GalNAc | *Maackia amurensis* |
| 43 | WGA | (GlcNAc)n and multivalent Sia | *Triticum unlgaris* |
| 44 | GSL-IA_4_ | α-GalNAc and GalNAcα-Thr/Ser (Tn) | *Griffonia simplicifolia* |
| 45 | GSL-IB_4_ | α-Gal | *Griffonia simplicifolia* |

Binding specificities are based on Lectin Frontier Database (LfDB; https://acgg.asia/lfdb2).

**Supplementary Table S2.** The Pearson correlation coefficients of the 45 lectin signals among the six LecChips.

|  | **LAA17100209** | **LAA17100210** | **LAA17100303** | **LAA17100307** | **LAA17100403** | **LAA17100404** |
| --- | --- | --- | --- | --- | --- | --- |
| LAA17100209 | 1 |  |  |  |  |  |
| LAA17100210 | 0.999** | 1 |  |  |  |  |
| LAA17100303 | 0.995** | 0.996** | 1 |  |  |  |
| LAA17100307 | 0.990** | 0.993** | 0.997** | 1 |  |  |
| LAA17100403 | 0.986** | 0.987** | 0.996** | 0.993** | 1 |  |
| LAA17100404 | 0.988** | 0.989** | 0.997** | 0.994** | 0.999** | 1 |

** *p* < 0.01.

**Supplementary Table S3.** Significant lectin signals between the noninvasive and invasive groups in discovery set.

|  | **Relative Intensity ^1^** | | ***p* ^2^** |
| --- | --- | --- | --- |
|  | **Noninvasive Group (*n* = 37)** | **Invasive Group (*n* = 55)** |  |
| LTL | 9.65 ± 4.01 | 7.38 ± 3.43 | 0.002 |
| PSA | 81.35 ± 10.48 | 76.27 ± 11.70 | 0.015 |
| AOL | 26.20 ± 3.27 | 24.71 ± 2.52 | 0.029 |
| SNA ^3^ | 56.26 ± 9.30 | 51.01 ± 8.48 | 0.010 |
| TJA-I | 69.68 ± 10.05 | 63.88 ± 7.66 | 0.004 |
| DSA ^3^ | 13.71 ± 13.26 | 8.86 ± 10.49 | 0.013 |
| NPA ^3^ | 63.17 ± 12.98 | 57.50 ± 10.86 | 0.040 |
| GNA ^3^ | 47.13 ± 9.38 | 40.68 ± 8.00 | 0.001 |
| HHL ^3^ | 21.32 ± 3.52 | 19.12 ± 3.93 | 0.015 |
| TJA-II | 12.66 ± 3.05 | 11.34 ± 2.64 | 0.027 |
| EEL ^3^ | 5.25 ± 4.46 | 3.40 ± 3.37 | 0.006 |
| STL | 17.72 ± 5.78 | 15.01 ± 5.19 | 0.015 |
| ACA | 26.16 ± 17.90 | 19.95 ± 15.34 | 0.009 |
| VVA ^3^ | 7.12 ± 2.31 | 5.48 ± 2.42 | 0.002 |
| Calsepa | 47.77 ± 3.43 | 49.42 ± 4.21 | 0.038 |
| PTL_I | 0.03 ± 0.10 | 0.06 ± 0.10 | 0.020 |
| WGA ^3^ | 13.65 ± 6.01 | 11.53 ± 6.15 | 0.036 |

^1^ Data are presented as mean ± SD unless indicated otherwise. ^2^ *p* values are derived from the Mann–Whitney U test between noninvasive lesions and invasive groups. ^3^ These lectins were validated to be significant both in discovery set and test set.
